# Efficacy of Biological Versus Synthetic Mesh in Ventral Hernia Repair: A Systematic Review and Meta-Analysis of Long-Term Outcomes and Recurrence Rates

**DOI:** 10.7759/cureus.92557

**Published:** 2025-09-17

**Authors:** Arif Khan, Sarah Elmadbouh, Sibthein A Khalid, Mohid Ayub, Sufyan Mahmood, Kapilraj Ravendran

**Affiliations:** 1 General Medicine, Wirral University Teaching Hospital, Wirral, GBR; 2 Surgery, Gradscape, London, GBR; 3 Spinal Surgery, Royal National Orthopaedic Hospital, London, GBR; 4 Surgery, Royal National Orthopaedic Hospital, London, GBR; 5 Surgery, NHS England, Leeds, GBR; 6 Surgery, Plovdiv Medical University, Plovdiv, BGR; 7 Surgery, East and North Hertfordshire NHS Trust, Stevenage, GBR

**Keywords:** abdomen ventral hernia, biologic mesh, characteristics of mesh, general and colorectal surgeon, laparoscopic ventral hernia repair, mesh repair, surgery, synthetic mesh

## Abstract

Ventral hernia repair commonly employs either biologic or synthetic mesh, yet the optimal choice remains debated. This systematic review and meta-analysis aimed to compare clinical outcomes such as infection, recurrence, and reoperation rates between biologic and synthetic mesh in ventral hernia repair.

Following Preferred Reporting Items for Systematic Reviews and Meta-Analyses (PRISMA) guidelines, a comprehensive literature search was conducted across MEDLINE, Cochrane Library, and EMBASE. Randomized controlled trials (RCTs) comparing biologic and synthetic mesh outcomes in adult ventral hernia repair were included. Risk of bias was assessed using the Risk of Bias 2 (ROBINS-II) tool. Pooled analyses used odds ratios (OR) for binary outcomes and weighted mean differences for continuous variables, with heterogeneity evaluated via I² statistics.

Four RCTs were included reporting outcomes for patients receiving biologic (n = 251-378) or synthetic mesh (n = 254-380). Recurrence rates were higher in the biologic mesh group (25.1%) compared to the synthetic group (12.7%) (OR: 2.30; 95% CI: 1.15-4.53; p = 0.01; I² = 73%). Additionally, reoperation rates were higher with biologic mesh (16.9%) versus synthetic mesh (10.4%), but this difference was not statistically significant (OR: 1.94; 95% CI: 1.06-2.44; p = 0.38; I² = 0%). Mesh infection incidence was also slightly higher with biologic mesh (4.8%) than with synthetic mesh (3.1%) (OR: 2.33; 95% CI: 0.64-3.73; p = 0.65; I² = 0%). However, no significant difference in mean patient age was observed between the groups (mean difference: -0.67; 95% CI: -2.78 to 1.44; p = 0.76; I² = 0%). Overall, biologic mesh was associated with a higher hernia recurrence compared to synthetic mesh, while differences in reoperation and mesh infection rates were not statistically significant.

These findings suggest that synthetic mesh offers more durable outcomes for ventral hernia repair. Biologic mesh may still be considered selectively in contaminated or high-risk surgical scenarios, but this recommendation is based on limited evidence. Further high-quality RCTs with longer follow-up are needed to better define the optimal mesh choice for varying clinical contexts.

## Introduction and background

Ventral hernias are defects in the abdominal wall through which intra-abdominal contents can protrude, typically occurring after surgery (incisional hernias). Still, they may also be primary (e.g., umbilical or epigastric hernias) [1]. They are common surgical problems, often requiring operative repair due to the risk of enlargement, discomfort, or strangulation of bowel [1]. These hernias result from a loss of integrity in the abdominal wall, either at sites of previous incisions or at naturally weaker areas. Incisional hernias are particularly common, reported in up to 20% of patients after laparotomy, whereas primary ventral hernias arise without prior surgical history and are often linked to congenital weakness, obesity, or increased intra‑abdominal pressure [2,3]. If untreated, they can significantly impair quality of life and may lead to serious complications, underscoring the importance of timely recognition and repair [2,3].

The use of prosthetic mesh has significantly reduced recurrence rates compared to suture repair alone [4]. Mesh reinforces the abdominal wall and promotes tissue integration [5]. Biologic mesh, derived from decellularized human or animal tissue, has been used since the early 2000s for complex or contaminated hernias [6,7], providing a collagen scaffold that supports integration and may reduce foreign body response and infection risk compared to synthetic meshes [8]. Biologic mesh undergoes gradual remodeling and resorption, allowing for the replacement of the scaffold with native tissue over time. This property can be advantageous in contaminated or high-risk surgical fields, potentially reducing long-term complications associated with permanent foreign materials [9]. The choice between biologic and synthetic meshes depends on patient factors and surgical context [9].

Synthetic mesh, made from non-absorbable materials like polypropylene or polyester, has been the standard for hernia repair since the 1950s due to its strength and durability [10,11]. Designed to be infection-resistant and biocompatible, it can be non-absorbable (e.g., polypropylene, polytetrafluoroethylene (PTFE)) for permanent support or absorbable for temporary reinforcement [12]. However, synthetic meshes may cause prolonged inflammation and complications such as pain, adhesions, infection, and contraction. Hybrid meshes combining synthetic and biologic materials have been developed to improve integration and reduce adverse reactions while maintaining strength [13].

## Review

Methodology

Search Strategy

The protocol for this systematic review and meta-analysis was developed and approved by all authors and conducted in accordance with the Preferred Reporting Items for Systematic Reviews and Meta-Analyses (PRISMA) guidelines [14]. Risk of bias was assessed independently by reviewers, with discrepancies resolved through consensus discussions. A comprehensive literature search was performed across multiple databases, including MEDLINE (via PubMed), Cochrane Library, and EMBASE. The following search terms were used in various combinations: ("Ventral Hernia" OR "Incisional Hernia" OR "Abdominal Wall Hernia") AND ("Biologic Mesh" OR "Biological Mesh" OR "Acellular Dermal Matrix" OR "Porcine Mesh" OR "Human-derived Mesh") AND ("Synthetic Mesh" OR "Polypropylene Mesh" OR "Polyester Mesh" OR "Composite Mesh") AND ("Hernia Recurrence" OR "Surgical Site Infection" OR "Wound Complication" OR "Seroma" OR "Postoperative Complications" OR "Morbidity" OR "Mortality" OR "Long-Term Outcomes"). Medical Subject Headings (MeSH) were applied where appropriate to enhance the search sensitivity.

Study Selection and Eligibility Criteria 

This meta-analysis included studies that met the following eligibility criteria: adults undergoing ventral hernia repair using either biological or synthetic mesh. Both randomized controlled trials (RCTs) and non-randomized comparative studies were considered. Studies were excluded if they involved pediatric populations or were systematic reviews, editorials, case reports, or conference abstracts. Only articles published in English with full-text availability were included. The primary focus was on studies reporting relevant clinical outcomes comparing biological and synthetic mesh in ventral hernia repair.

The baseline characteristics extracted included patient demographics, surgical techniques, mesh type, and operative indications. Both short-term postoperative complications and long-term outcomes, such as hernia recurrence, were examined.

The review protocol was registered at PROSPERO under registration number CRD420251090386.

Data Extraction and Quality Assessment 

Based on predetermined criteria, three researchers (AK, SE, SK) extracted data, and one senior researcher (KR) reviewed and verified the outcomes. Extracted data included publication date, study design and type, year and country of study, median patient age, recurrence rates, mesh infection rates, reoperation rates, quality of life (QOL) measures, main limitations, and implications.

Risk of bias was assessed by two authors (AK, SK) using the Risk of Bias 2 (ROBINS-II) tool [15]. This tool evaluates potential biases, including confounding, participant selection, intervention classification, deviations from intended interventions, outcome measurement, and selective reporting. Statistical analyses were performed using IBM SPSS Statistics for Windows, version 28.0 (IBM Corp., Armonk, USA), and funnel plots were used to assess publication bias.

Statistical Analyses

This meta-analysis was conducted following the Cochrane Collaboration guidelines and the PRISMA statement [14,16]. Dichotomous data were analyzed using the Mantel-Haenszel method to calculate odds ratios (OR) with 95% CI, while continuous outcomes were pooled using weighted mean differences. Statistical heterogeneity was assessed using the Cochran’s Q test (Chi-squared) and the I² statistic, with a p-value < 0.10 and an I² value > 25% considered indicative of significant heterogeneity. All analyses were performed using Review Manager (RevMan), version 5.4 (The Cochrane Collaboration, London, UK).

Results 

**Figure 1:**
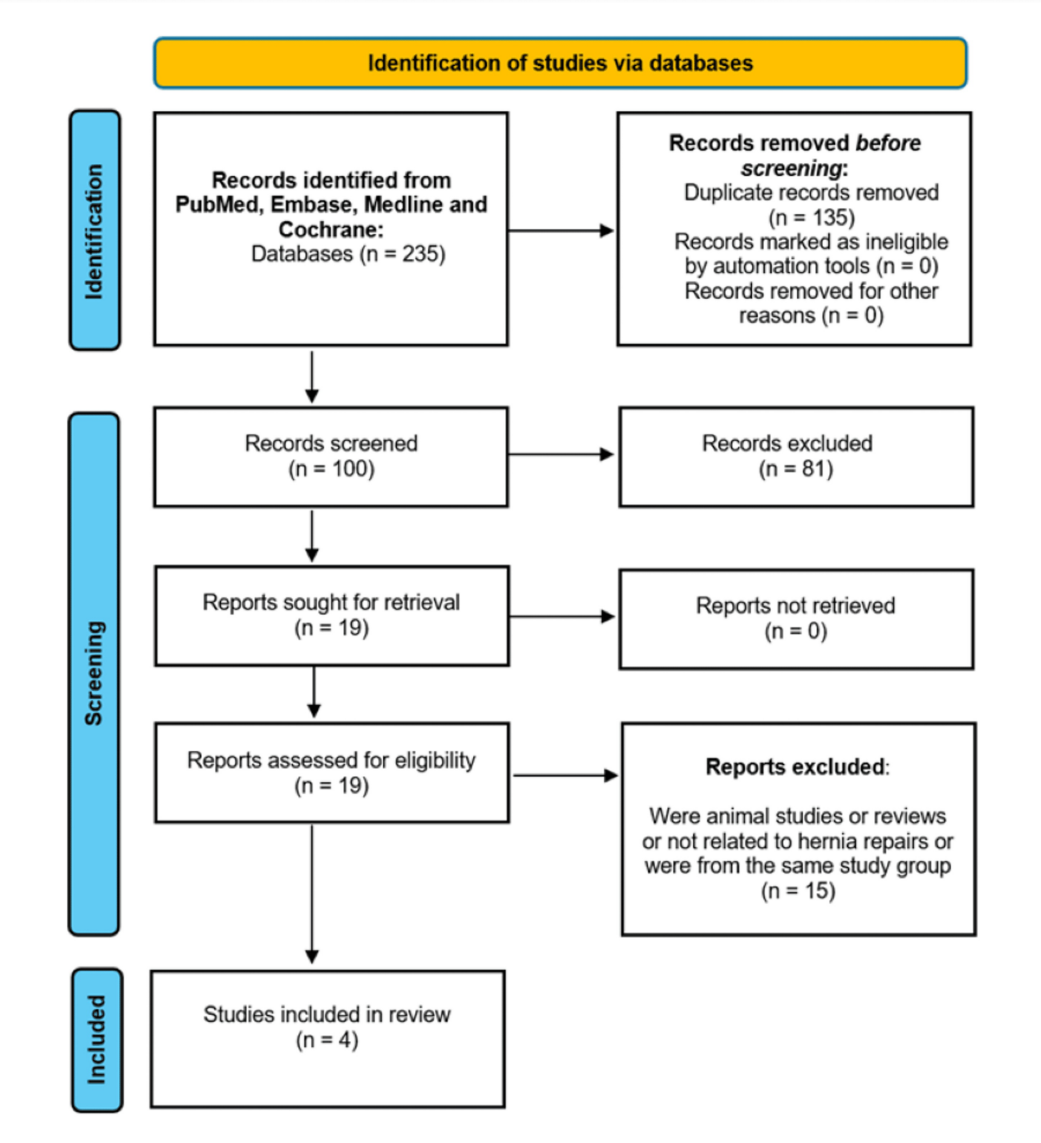
PRISMA flow diagram demonstrating the literature selection strategy. PRISMA: Preferred Reporting Items for Systematic Reviews and Meta-Analyses

A total of 235 records were identified through the initial database search, of which 135 were removed as duplicates. After duplicate removal, 100 records remained, and 81 were excluded during abstract screening based on the population, intervention, comparison, and outcome (PICO) criteria. Full-text screening was then performed on the remaining 19 articles, with 15 being excluded after applying the eligibility criteria. Overall, four studies were included in the final analysis. A PRISMA flow diagram illustrating the study selection process is presented in Figure 1 [14].

From the four included RCTs, outcomes were reported for patients undergoing ventral hernia repair with either biological or synthetic mesh [17-20]. Recurrence rates were higher in the biological mesh group, with 93 of 378 patients (24.6%) experiencing recurrence compared to 37 of 380 patients (9.7%) in the synthetic group. Mesh infection occurred in 12 of 251 patients (4.8%) receiving biological mesh versus eight of 254 patients (3.1%) with synthetic mesh. Reoperation rates, reported in three studies, were also higher for biological mesh (50 of 296 patients; 16.9%) compared to synthetic mesh (31 of 297 patients; 10.4%). Table 1 summarizes the overall findings from the included studies.

**Table 1:** Overall findings from the included studies, comprising five publications comparing clinical outcomes of biologic versus synthetic mesh in ventral hernia repair. * QOL not commented on. ** Median - mean value and standard deviation not given. *** Mesh infection not commented on. QOL: Quality of life; AW-QOL: Abdominal wall quality of life; VHR: Ventral hernia repair; HerQles: Hernia-related quality of life survey; EQ-5D: EuroQol 5-dimension questionnaire; RCT: Randomized controlled trial

Author	Publication Date	Type of Study	Year of Study	Country	Mean Age ± SD - Biologic Mesh	Mean Age ± SD - Synthetic Mesh	Recurrence Rates - Biologic Mesh	Recurrence Rates - Synthetic Mesh	Mesh Infection - Biologic Mesh	Mesh Infection - Synthetic Mesh	Reoperation Rates - Biologic Mesh	Reoperation Rates - Synthetic Mesh	Quality of Life
Harris et al. [17]	04/2021	RCT	03/2014-10/2018	USA	55.0 ± 11.5	55.5 ± 11.1	32 (39% )	18 (22%)	5 (6.1%)	4 (4.8%)	*	*	*
Miserez et al. [18]	01/2021	RCT	01/09/2005-07/08/2009	Europe	58.5 ± 13.8	60.1 ± 12.6	25 (20%)	7 (5%)	2 (2%)	2 (2%)	22 (17%)	9 (7%)	-
Olavarria et al.‌ [19]	06/2021	RCT	09/2017-01/2019	USA	51 ± 9.9	51 ± 12.1	10 (30.3%)	5 (13.5%)	5 (15.2%)	2 (5.4%)	4 (12%)	3 (8%)	At one-year follow-up, mean AW-QOL scores of patients who underwent complex open VHR with biologic mesh improved by 20.3 points, whereas on average those in the synthetic mesh group experienced an increase of 27.9 points.
Rosen et al. [20]	01/2022	RCT	12/2012-04/2019	USA	(63.7***)	(63.7**)	26 (20.5%)	7 (5.6%)	***	***	24 (19%)	19 (15%)	Patients had similar baseline QOL measurements, with overall improvements in both groups. There were no significant differences between the groups regarding QOL measured by EQ-5D and HerQles scores at any time point.

Mean Age

**Figure 2:**
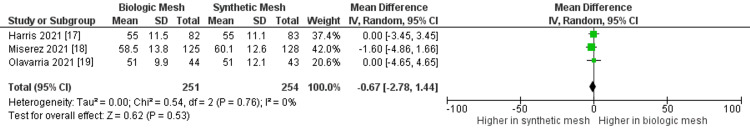
Meta-analysis comparing mean age between patients undergoing biologic and synthetic mesh repair.

**Figure 3:**
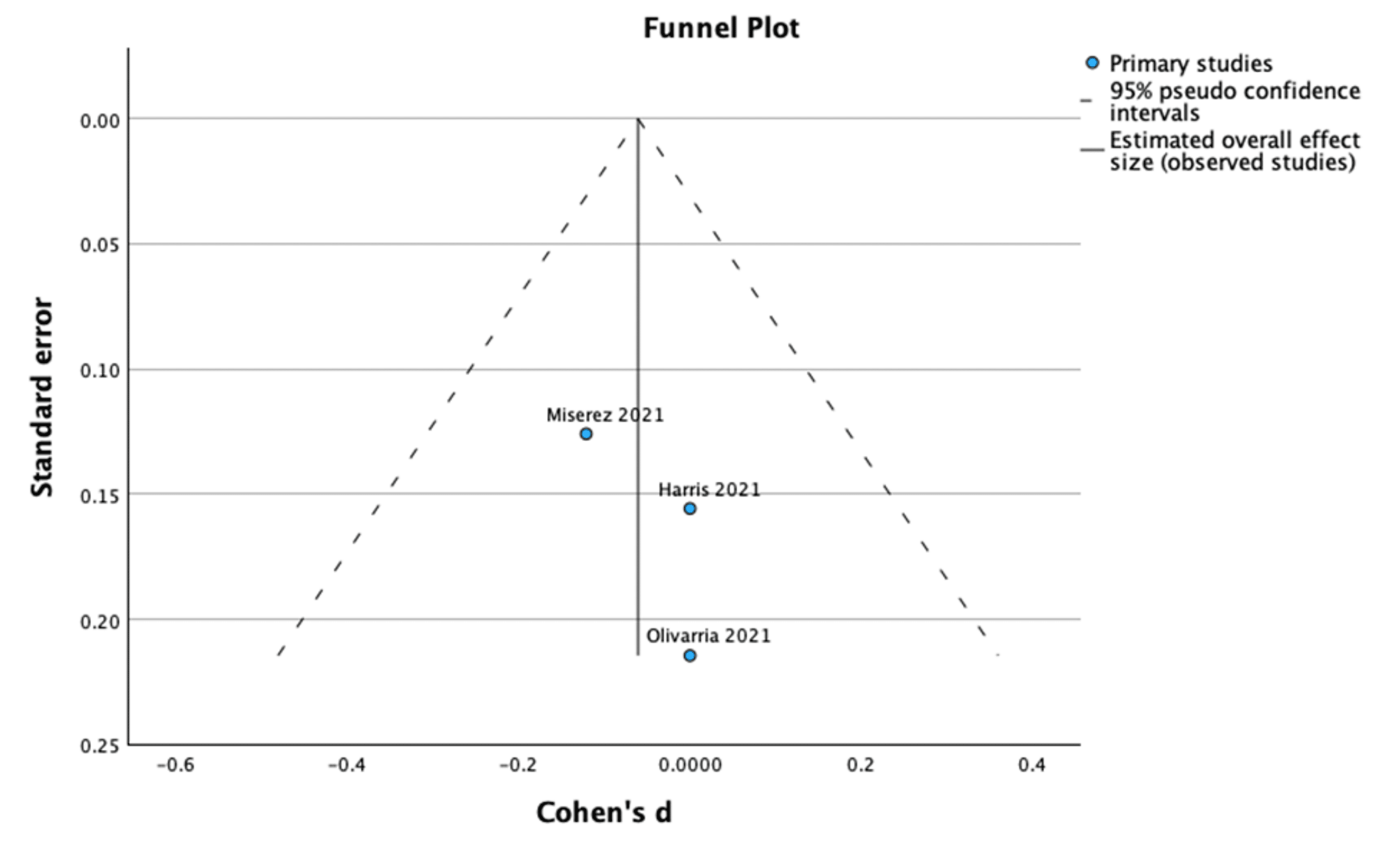
Funnel plot and Egger’s test for median age. The funnel plot and Egger’s test for mean age showed no evidence of significant publication bias (Effect Size = -0.061; Standard Error = 0.089; Z = -0.681; p = 0.496; 95% CI: -0.235 to 0.114).

Our analysis demonstrated no significant difference in mean age between patients receiving biological mesh and those receiving synthetic mesh (mean difference: -0.67; 95% CI: -2.78 to 1.44; p = 0.76; I² = 0%), as shown in Figure 2 [17-20]. Figure 3 shows the funnel plot and Egger’s test for mean age.

Mesh Infection

**Figure 4:**
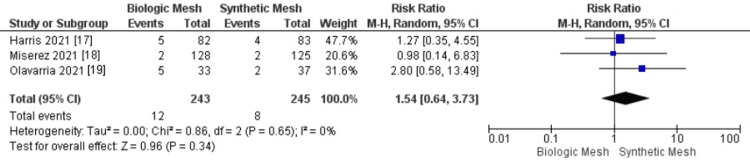
Meta-analysis comparing mesh infection rates between biologic and synthetic mesh in ventral hernia repair.

**Figure 5:**
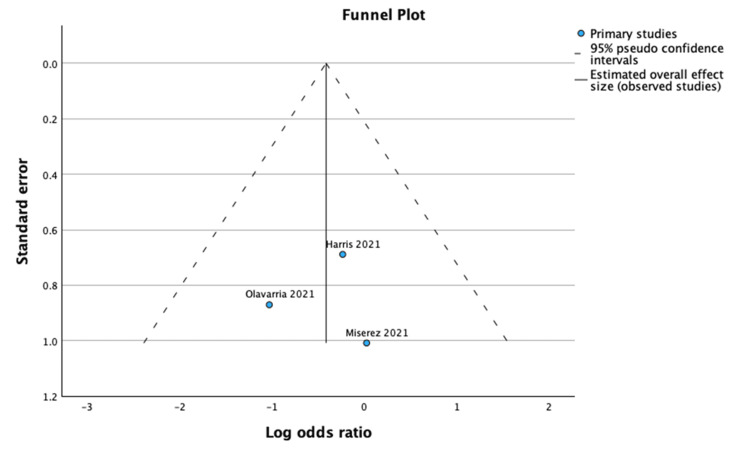
Funnel plot and Egger’s test for mesh infection. The funnel plot and Egger’s test for mesh infection showed no evidence of significant publication bias (Effect Size = -0.415; Standard Error = 0.476; Z = -0.873; p = 0.383; 95% CI: -1.348 to 0.517).

Our analysis showed a higher incidence of mesh infection in the biologic mesh group compared to the synthetic mesh group; however, this difference did not reach statistical significance (95% CI: 0.64-3.73; p = 0.65; I² = 0%), as demonstrated in Figure 4 [17-20]. Figure 5 shows the funnel plot and Egger’s test for mesh infection.

Recurrence Rates 

**Figure 6:**
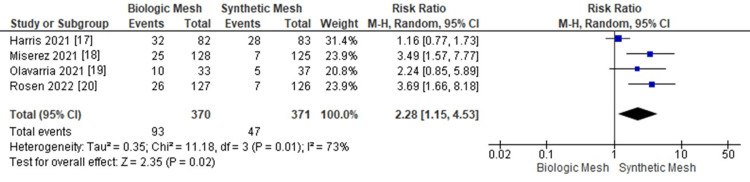
Meta-analysis comparing hernia recurrence rates between biologic and synthetic mesh in ventral hernia repair.

**Figure 7:**
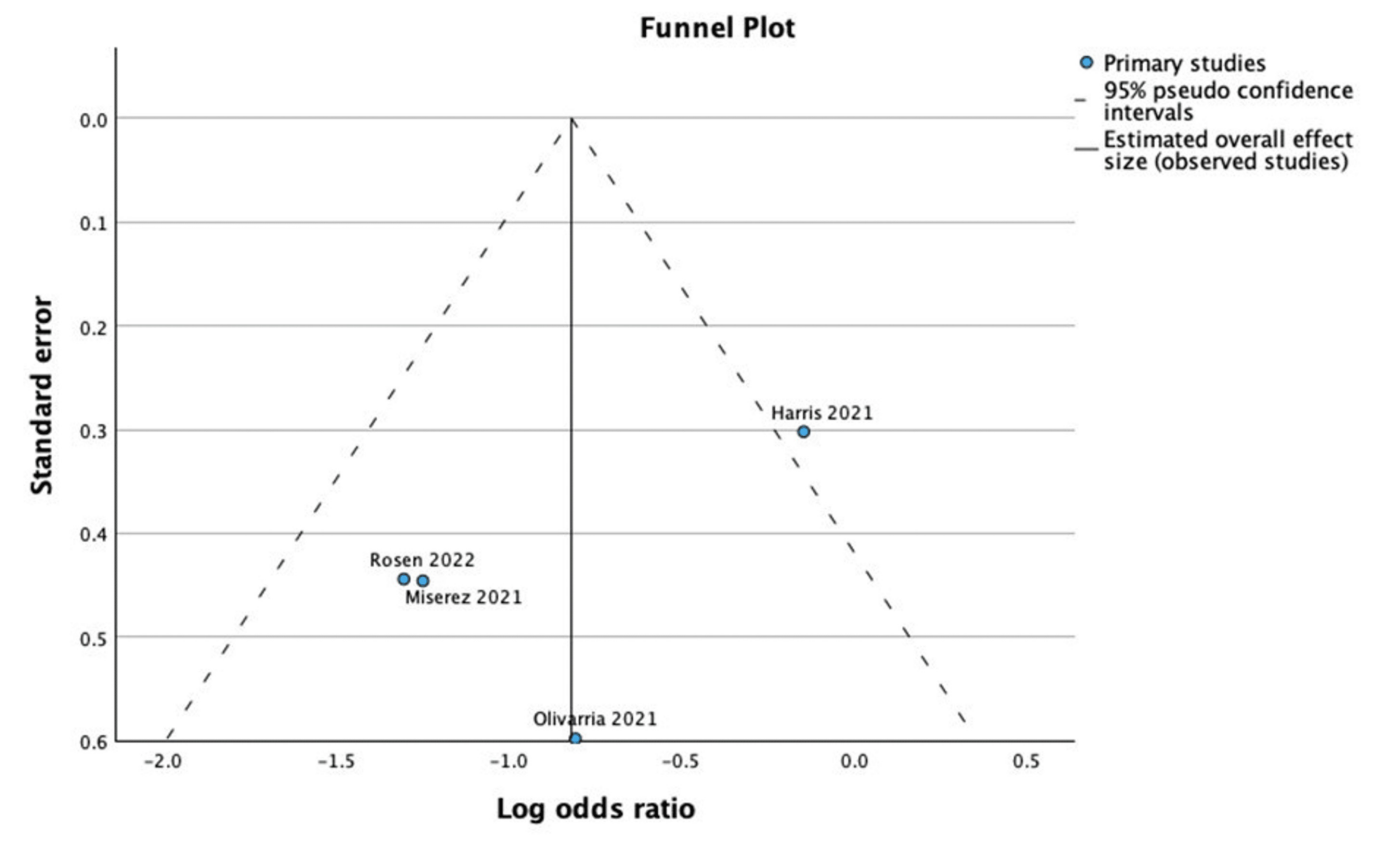
Funnel plot and Egger’s test for recurrence rates. The funnel plot and Egger’s test for recurrence rates suggested possible publication bias (Effect Size = -0.819; Standard Error = 0.319; Z = -2.568; p = 0.010; 95% CI: -1.444 to -0.194).

Analysis demonstrated that patients who underwent ventral hernia repair with biologic mesh had significantly higher recurrence rates compared to those with synthetic mesh (95% CI: 1.15-4.53; Z = 2.35; p = 0.01; I² = 73%), as shown in Figure 6 [17-20]. Figure 7 demonstrates the funnel plot and Egger’s test for recurrence rates.

Reoperation Rates 

**Figure 8:**
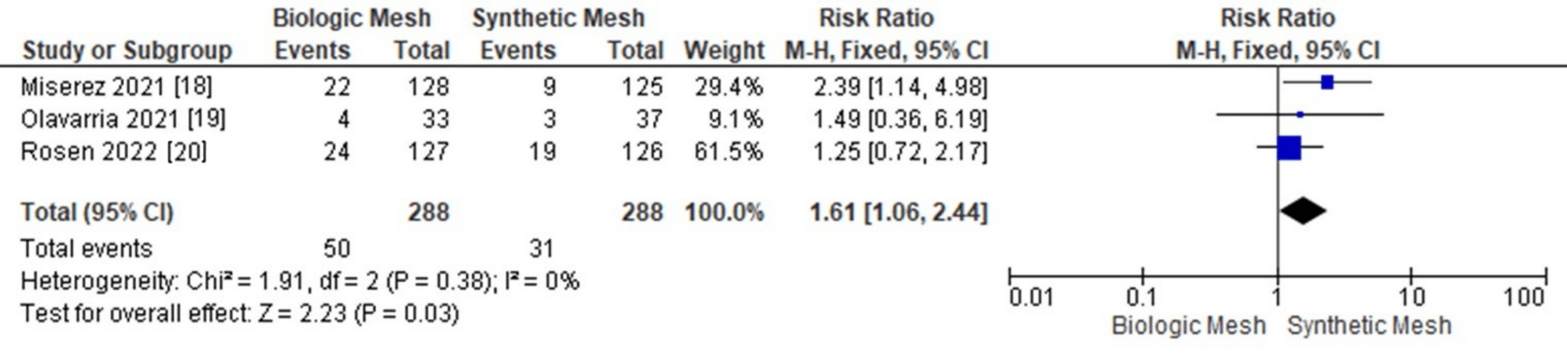
Meta-analysis comparing reoperation rates between biologic and synthetic mesh in ventral hernia repair.

**Figure 9:**
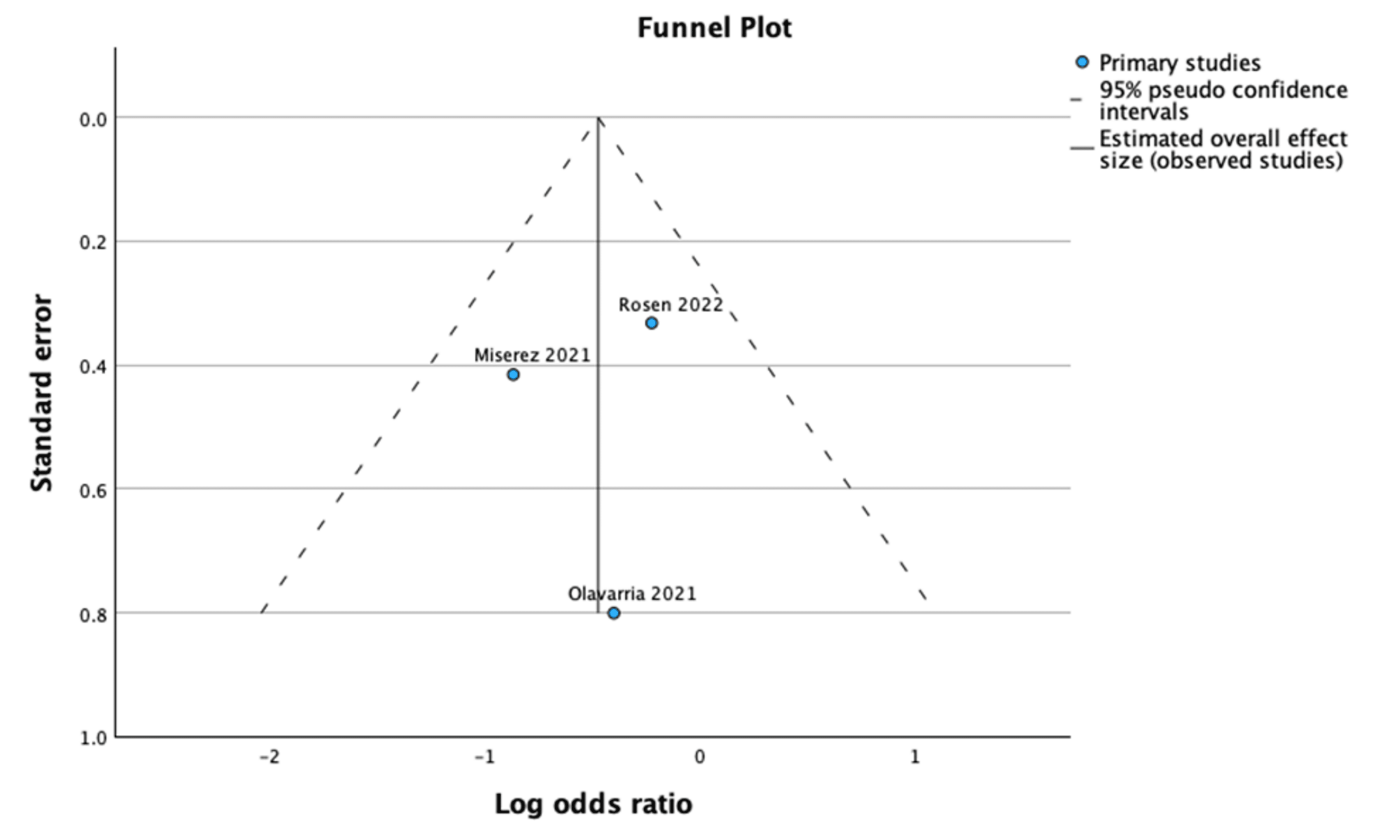
Funnel plot and Egger’s test for reoperation rates. The funnel plot and Egger’s test (p = 0.066) showed no significant publication bias in this meta-analysis.

Biologic mesh was associated with a higher reoperation rate compared to synthetic mesh; however, this difference did not reach statistical significance (OR: 1.94; 95% CI: 1.06 - 2.44; p = 0.38; I² = 0%), as shown in Figure 8 [17-20]. Figure 9 demonstrates the funnel plot and Egger’s test for reoperation rates.

Discussion

In the current literature, both synthetic and biologic meshes have been extensively studied for complex ventral hernia repair. Our meta-analysis demonstrates that synthetic mesh is associated with a lower recurrence rate compared to biologic mesh, consistent with previous reports highlighting the superior durability and lower failure rates of synthetic materials in hernia repair [4,7,17]. Specifically, 25.1% of patients receiving biologic mesh experienced recurrence compared to 12.7% in the synthetic group (95% CI: 1.15-4.53; p = 0.01; I² = 73%). These findings underscore the longer-term durability of synthetic mesh, a result corroborated by earlier systematic reviews and RCTs [21-23]. Reoperation rates were higher in patients with biologic mesh (17.3%) compared to synthetic mesh (10.7%), although this difference did not reach statistical significance (95% CI: 1.06-2.44; p = 0.38; I² = 0%). These results suggest that while reoperation may be more frequent with biologic mesh, the evidence does not conclusively demonstrate a statistically significant difference in this outcome [17-20].

Our meta-analysis also showed a higher but non-significant incidence of mesh infection with biologic mesh (4.9%) compared to synthetic mesh (3.1%) (95% CI: 0.64-3.73; p = 0.65; I² = 0%). This aligns with prior reports, such as Lorenz et al. [24], which found comparable infection rates between biologic (1.2%) and synthetic mesh (0.9%) in a propensity-matched cohort of 519 patients. These findings suggest that when patient and wound factors are controlled, infection rates may not differ significantly between mesh types.

Two of the included studies reported on QOL outcomes following ventral hernia repair [19,20]. Improvements in QOL and pain scores were observed over time, regardless of mesh type. One study reported a 20.3-point improvement in abdominal wall quality of life (AW-QOL) scores at one year for patients receiving biologic mesh versus a 27.9-point increase in the synthetic mesh group [19]. These results indicate that both mesh types can achieve meaningful QOL improvements, although patient-specific factors such as hernia size, obesity, tobacco use, and prior surgeries may influence outcomes [25].

Recent innovations, including hybrid meshes combining biosynthetic and biologic components, have shown early promise in reducing surgical site occurrences while maintaining low recurrence rates, even in older or comorbid patients [23]. These developments highlight the potential for new mesh technologies to mitigate current limitations associated with both synthetic and biologic meshes.

While synthetic mesh demonstrates superior durability with lower recurrence rates, biologic mesh may still be appropriate in select patients, particularly those with contaminated or high-risk wounds where infection risk is elevated. However, this recommendation is based on limited evidence and should be interpreted cautiously. Clinical judgment and patient-specific factors should guide mesh selection to optimize outcomes and QOL.

Limitations 

The studies included in this analysis were limited by a small number of RCTs and exhibited variability in patient populations, follow-up durations, and surgical techniques, which may influence the consistency of outcomes. Differences in mesh placement, fixation methods, and perioperative care add further heterogeneity. Additionally, funnel plot and Egger’s test analyses suggested possible publication bias for recurrence rates. Despite these limitations, the overall findings provide insights into the relative efficacy and safety profiles of biologic and synthetic meshes in ventral hernia repair.

## Conclusions

This meta-analysis demonstrates that biologic mesh is associated with higher recurrence rates compared to synthetic mesh in ventral hernia repair, while differences in reoperation and mesh infection rates did not reach statistical significance. Synthetic mesh remains the more durable option for most patients, offering a favorable balance of long-term outcomes and safety. Biologic mesh may still be appropriate in select patients, particularly those with contaminated or high-risk wounds, but this recommendation is based on limited evidence. These findings underscore the importance of individualized mesh selection and highlight the need for future well-designed RCTs with larger cohorts and longer follow-up to optimize clinical decision-making.
